# Comprehensive Quantitative Analysis of 32 Chemical Ingredients of a Chinese Patented Drug Sanhuang Tablet

**DOI:** 10.3390/molecules22010111

**Published:** 2017-01-12

**Authors:** Hau-Yee Fung, Yan Lang, Hing-Man Ho, Tin-Long Wong, Dik-Lung Ma, Chung-Hang Leung, Quan-Bin Han

**Affiliations:** 1 School of Chinese Medicine, Hong Kong Baptist University, Hong Kong, China; 15485862@life.hkbu.edu.hk (H.-Y.F.); alanhhm@hkbu.edu.hk (H.-M.H.); 15485021@life.hkbu.edu.hk (T.-L.W.); 2 First Affiliated Hospital, Zhengzhou University, Zhengzhou 450001, China; graduately@163.com; 3 Department of Chemistry, Hong Kong Baptist University, Hong Kong, China; edmondma@hkbu.edu.hk; 4 State Key Laboratory of Quality Research in Chinese Medicine, Institute of Chinese Medical Sciences, University of Macau, Macao, China; duncanleung@umac.mo

**Keywords:** sanhuang tablet, Chinese patented drug, quantitative analysis, quality evaluation, CAD

## Abstract

Sanhuang Tablet (SHT) is a Chinese patented drug commonly used for the treatment of inflammations of the respiratory tract, gastrointestinal tract, and skin. It contains a special medicinal composition including the single compound berberine hydrochloride, extracts of Scutellariae Radix and Rhei Radix et Rhizoma, as well as the powder of Rhei Radix et Rhizoma. Despite advances in analytical techniques, quantitative evaluation of a Chinese patented drug like SHT remains a challenge due to the complexity of its chemical profile. In this study, ultra-high performance liquid chromatography coupled with quadrupole-time-of-flight mass spectrometry (UHPLC-Q-TOF-MS) was used to simultaneously quantify 29 non-sugar small molecule components of SHT (11 flavonoids, two isoflavonoids, one flavanone, five anthraquinones, two dianthranones, five alkaloids, two organic acids and one stilbene). Three major saccharide components, namely fructose, glucose, and sucrose, were also quantitatively determined using high performance liquid chromatography-charged aerosol detector (HPLC-CAD) on an Asahipak NH_2_P-50 4E amino column. The established methods were validated in terms of linearity, sensitivity, precision, accuracy, and stability, and then successfully applied to analyze 27 batches of commercial SHT products. A total of up to 57.61% (*w*/*w*) of SHT could be quantified, in which the contents of the determined non-saccharide small molecules varied from 5.91% to 16.83% (*w*/*w*) and three saccharides accounted for 4.41% to 48.05% (*w*/*w*). The results showed that the quality of the commercial products was inconsistent, and only four of those met Chinese Pharmacopoeia criteria.

## 1. Introduction

Sanhuang tablet (*Sanhuang Pian* in Chinese, SHT), comprised of the powder of Rhei Radix et Rhizoma (dried root and rhizome of *Rheum palmatum* L., *R. tanguticum* Maxim. Ex Balf, or *R. officinale* Baill.), berberine hydrochloride, and extracts of Scutellariae Radix (dried root of *Scutellaria baicalensis* Georgi) is a commonly used Chinese patented drug [1]. SHT is widely used in the treatment of constipation, gastroenteritis, dysentery, and vaginitis [2,3,4]. According to the public access database on the website of the China Food and Drug Administration, as of September 2016, SHT, with 210 licenses, is one of the top 25 licensed Chinese patented drugs in China. Meanwhile, Health Canada has licensed four SHT products as natural health products.

Different from conventional Chinese medicine preparations, SHT is not a mixture of herb extracts, but rather a mixture of a single chemical component, two herb extracts, and a powdered raw herb. This formula is derived from Sanhuang Xiexin decoction, which consists of Coptidis Rhizoma (Dried rhizome of *Coptis chinensis* Franch, *C. deltoidea* C. Y. Cheng et Hsiao, or *C. teeta* Wall), Rhei Radix et Rhizoma, and Scutellariae Radix. As recorded in the currently available literature [5,6,7], this formula was first mentioned in *Synopsis of Prescriptions of the Golden Chamber*, written in the Eastern Han dynasty (A.D. 25–220) by Zhang Zhong-Jing. Different dosage forms, such as honey bolus and powder, were invented in the Tang dynasty (618–907). The honey bolus was officially recognized in the Song dynasty (960–1279) when the Bureau of Imperial Physicians recorded it in an official formulary, *Prescriptions from the Great Peace Imperial Grace Pharmacy*. Its tablet preparation was developed in 1958 when Cotidis Rhizoma was replaced with its main component, berberine hydrochloride, and Scutellariae Radix was replaced with its extract.

According to the chemical profiles, SHT is more complex than Sanhuang Xiexin decoction. Raw, powdered Rhei Radix et Rhizoma contributes a chemical diversity that includes anthraquinones and their glycosides, anthrones and their glycosides, stilbenes, polysaccharides, tannins, and some organic tissues of the plant [8]. The extract of Scutellariae Radix mainly contains flavonoids [9], especially when the water extraction and subsequent acid precipitation heightens the content of baicalin [10]. Furthermore, the berberine hydrochloride that represents Coptidis Rhizoma is supposed to be a single compound. However, it is always accompanied by several analogues due to inherent difficulties in separating single components from a natural product.

SHT has the pharmacological activities of anti-inflammatory, anti-bacterial and purgation [2,3,11]. The purgative, antibacterial and antivirus activities of Rhei Radix et Rhizoma make it become the principal drug of the formulation. Anthrones, such as sennoside, rheidin, and palmidin, are responsible for the purgative activity [12]. The free-anthraquinone derivatives provide inhibition effect on Helicobacter pylori, which is one of the causes of inflammation and ulcer in stomach and duodenum [13]. Aloe-emodin has inactivation effects on varicella zoster virus, pseudorabies virus and influenza virus [13] and an anti-inflammatory effect [14]. The active components of Scutellariae Radix are flavonoids. Their bacteriostatic, antipyretic and analgesic activities are strongly related to the action of Sanhuang tablet [15]. Meanwhile, berberine hydrochloride itself is considered as an antibacterial drug effective against *Staphylococus aureus* and *Enterococcus faecalis* [16].

Despite the great advances in analytical tools, the quality control of Chinese medicines, especially in terms of quantitative analysis of the chemical profile, remains unsatisfactory. On the other hand, the successful licensing of botanical drugs, like VEREGEN, by the FDA has highlighted the importance of quantitative analysis of natural complexes like Chinese medicines. In the case of SHT, the published quantitative analysis has assessed no more than ten compounds [17,18,19], missing many important ingredients. For example, sennoside A, a major component responsible for the purgative action of Rhei Radix et Rhizoma [20,21] and an assay marker of the herb in the Japanese Pharmacopoeia and Korea Pharmacopoeia [22,23], has not been quantified in SHT by the aforementioned studies nor the Chinese Pharmacopoeia. Furthermore, SHT also contains plenty of carbohydrates such as monosaccharides, oligosaccharides, and polysaccharides; however, the analytical methods published to date for the analysis of SHT have not determined either the identity or amount of the carbohydrates. This study aims to establish a more comprehensive analytical method to quantitatively analyze the chemical profile of SHT. Such an approach will be useful not only in determining the quality of various commercial SHT products but also in assessing other, similarly complex, herbal preparations.

## 2. Results and Discussion

### 2.1. Identification of Chemical Components in SHT Samples

**Figure 1:**
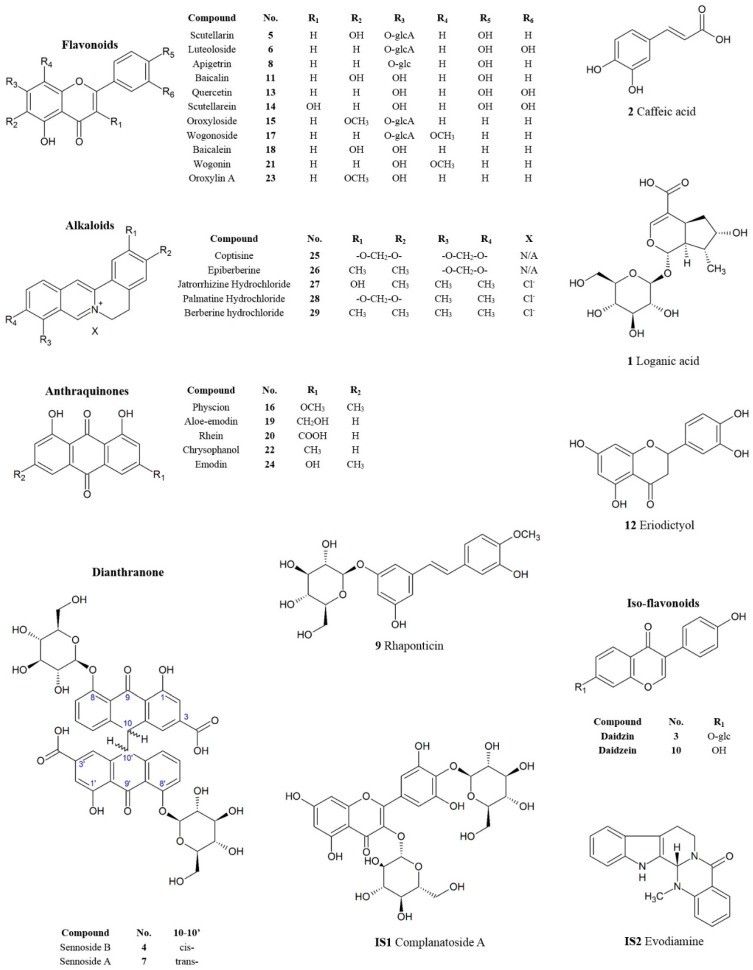
Chemical structures of the 32 quantitatively analyzed components and internal standards.

There are three major groups of chemicals in SHT samples: flavonoids, anthraquinones, and alkaloids. The chemicals gave clear signals under negative ion mode in MS analysis except alkaloids which were examined using positive ion mode. Complanatoside A (**IS1**) and evodiamine (**IS2**) were used as internal standards as they share similar chemical structures with the analytes but with different retention time. After comparison of the retention times and MS data with those of chemical reference standards, 32 compounds were identified in SHT commercial samples. Among these 32 compounds, there were 11 flavonoids, two isoflavonoids, one flavanone, five anthraquinones, two dianthranones, five alkaloids, two organic acids, one stilbene, and three saccharides. Their chemical structures are given in Figure 1.

**Figure 2:**
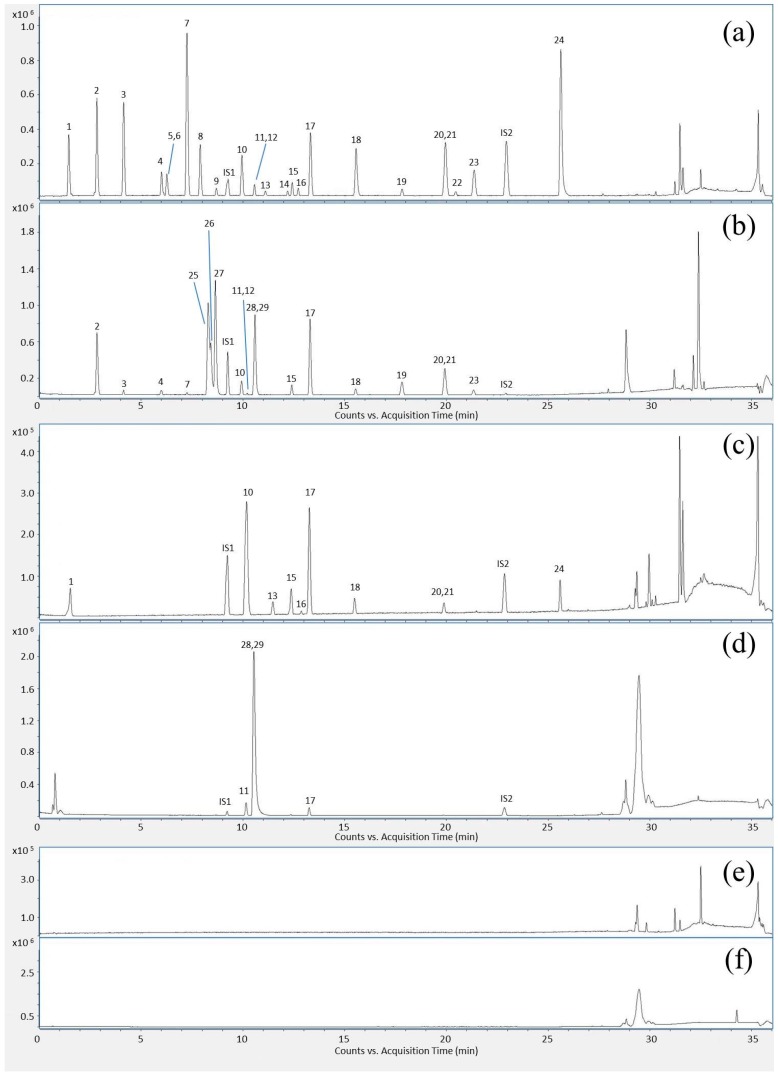
BPC of mixed reference standards under (**a**) negative ion mode and (**b**) positive ion mode; BPC of typical SHT sample under (**c**) negative ion mode and (**d**) positive ion mode; BPC of ethanol–water solution (70:30, *v*/*v*) under (**e**) negative ion mode and (**f**) positive ion mode.

**Figure 3:**
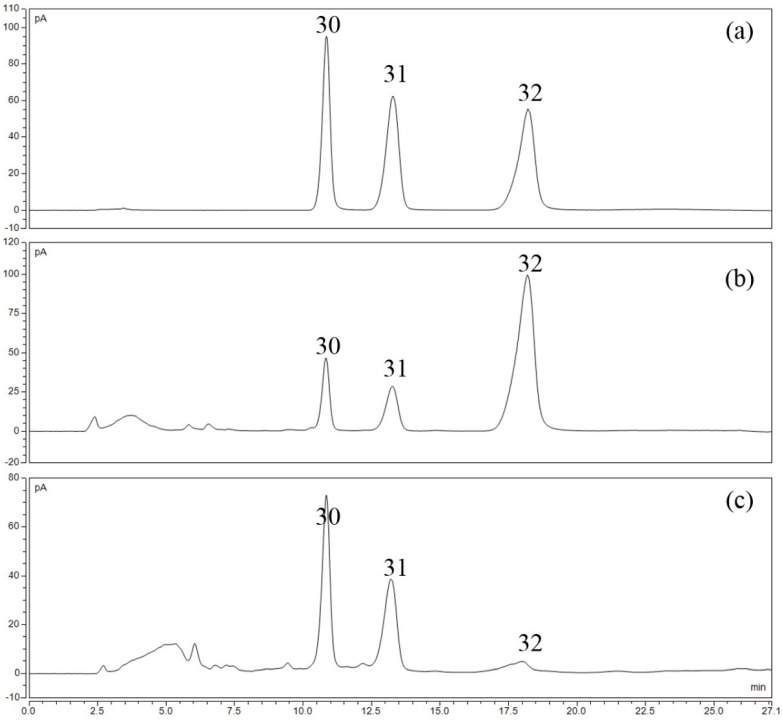
HPLC-CAD chromatogram of (**a**) mixed reference standards; (**b**) typical sugar -coated SHT sample and (**c**) self-made control sample.

Figure 2a shows the BPC of 24 mixed standards in negative ion mode: (**1**) loganic acid; (**2**) caffeic acid; (**3**) daidzin; (**4**) sennoside B; (**5**) scutellarin; (**6**) luteoloside; (**7**) sennoside A; (**8**) apigetrin; (**9**) rhaponticin; (**10**) daidzein; (**11**) baicalin; (**12**) eriodictyol; (**13**) quercetin; (**14**) scutellarein; (**15**) oroxyloside; (**16**) physcion; (**17**) wogonoside; (**18**) baicalein; (**19**) aloe-emodin; (**20**) rhein; (**21**) wogonin; (**22**) chrysophanol; (**23**) oroxylin A and (**24**) emodin. Figure 2b shows the BPC of five compounds under positive ion mode: (**25**) coptisine; (**26**) epiberberine; (**27**) jatrorrhizine hydrochloride; (**28**) palmatine hydrochloride and (**29**) berberine hydrochloride. Three saccharides were identified as (**30**) fructose; (**31**) glucose; and (**32**) sucrose by HPLC-CAD, as showed in Figure 3.

### 2.2. Method Validation

As shown in Table 1, for the UHPLC-Q-TOF-MS method, all the calibration curves of the 29 analytes showed good linearity with coefficients (R^2^) no less than 0.99. All the LOQs and LODs were at the nanogram level. Relative standard deviations (RSDs) of intra-day precision, inter-day precision, and stability were all less than 5%, indicating the good precision of method and sample stability. The established method also gave an acceptable accuracy with a spike recovery of 95%–105% for all analytes. For the HPLC-CAD method, a good linearity was demonstrated. The LODs and LOQs of the three saccharides were at microgram level. RSDs of the intra-day precision, inter-day precision and stability were all less than 5% while the accuracy was within 94%–105% of the actual values. These results indicated a satisfactory reliability and an accuracy of these two developed methods for the quantification of 32 analytes in SHT.

In our previously published HPLC-ELSD method [24], when using the similar chromatographic conditions and the same HPLC Asahipak NH_2_P-50 4E (4.6 × 250 mm) column, the LODs of fructose, glucose and sucrose were about 50, 30, and 10 µg/mL, respectively. In this study, the new CAD detector significantly decreased the LODs to 1.24, 1.84 and 2.27 µg/mL, respectively, suggesting that CAD is much more sensitive for the qualitative and quantitative analysis of saccharides.

### 2.3. Quantification of 32 Components in Commercial SHT Samples

**Figure 4:**
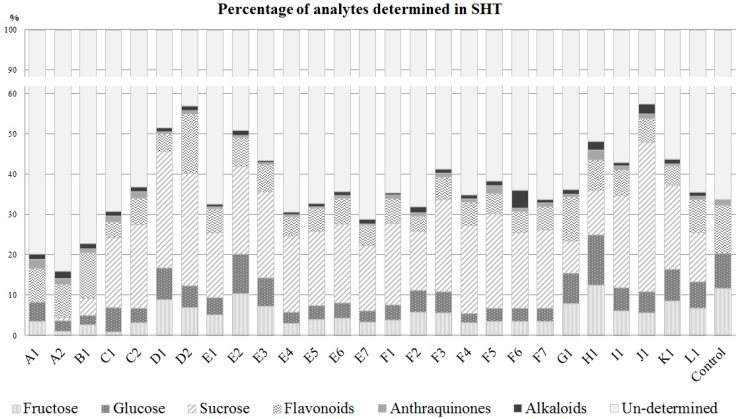
Percentage of chemical components quantified in SHT samples.

The established UHPLC-Q-TOF-MS and HPLC-CAD methods were successfully applied to the quantitative determination of 29 non-saccharide small molecules and three saccharides in 27 commercial SHT samples produced by 12 manufacturers. The results are shown in Table 2 and Figure 4. For the UHPLC-Q-TOF-MS, extracted ion chromatograms (EIC) were used for quantification. The 32 components accounted for 15.75%–57.61% (*w*/*w*) of the overall weight of the SHT samples, whereas 29 non-saccharide small molecules accounted for 5.91%–16.83%. Among these chemicals, baicalin, berberine hydrochloride, oroxyloside, emodin, and wogonoside were the major non-saccharide chemicals in the 27 SHT samples. The quantification percentages of two monosaccharides varied in the range of 3.55%–24.91%; and sucrose (7.92%–37.21%) is the major saccharide in sugar-coated SHT. The undetermined chemicals constituted 42.39%–84.25% of SHT which was attributable to the unknown tablet excipients. Another possible reason is the presence of non-extractable plant tissues of Rhei Radix et Rhizoma as its raw herb powder accounts for 46% of the total weight of SHT and only 27.69% of the powder is extractable in this study.

**Figure 5:**
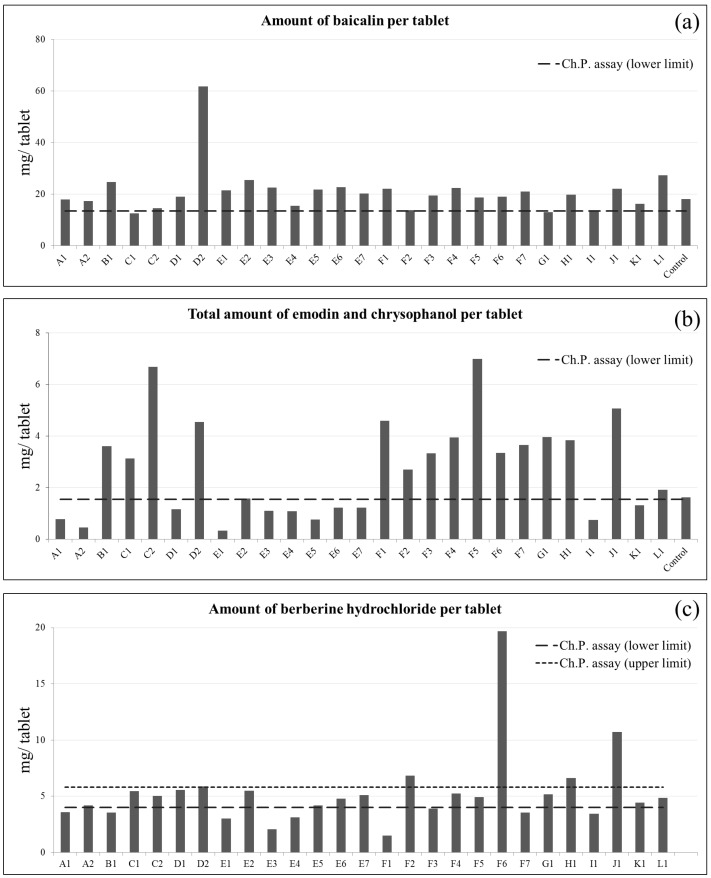
Results of commercial SHT samples under *Ch.P.* assay requirement for (**a**) amount of baicalin; (**b**) total amount of emodin and chrysophanol; and (**c**) amount of berberine hydrochloride per tablet.

The chemical profiles of commercial SHT samples were far from consistent and did not meet the assay requirements in *Ch.P. 2015*. Figure 5a shows the detected amount of baicalin per tablet, Figure 5b shows the total amount of emodin and chrysophanol per tablet, and Figure 5c shows the amount of berberine hydrochloride per tablet. Among 27 tested commercial samples, two contained less than 13.5 mg of baicalin per tablet and 11 contained less than 1.55 mg of the total amount of emodin and chrysophanol per tablet. The content of berberine hydrochloride should be within the range of 4.0–5.8 mg per tablet, but we found it below the level in nine samples and beyond the range in five samples. Only five out of 27 tested commercial samples fulfilled the assay requirements of *Ch.P. 2015*. In contrast to the commercial samples, the contents of baicalin and the total amount of emodin and chrysophanol in our control sample were 18.06 and 1.63 mg/tablet, respectively, which fulfilled the assay requirement in the *Ch.P. 2015*.

Other studies have similarly documented the inconsistent and unsatisfactory qualities of SHT on the market [17,18,19]. In those studies, the reported content of baicalin varied from 3.34 to 19.88 mg per tablet. The reported total amount of emodin and chrysophanol per tablet varied from 0.4 to 2.01 mg, while the content of berberine hydrochloride varied from 2.37 to 5.67 mg per tablet. The variations may come from the diversity of herbal origin, manufacturers’ production protocols, the process of sample pretreatment before analysis or storage conditions.

*Ch.P.* states that only the dried root and rhizome of three species, namely *Rheum palmatum* L., *R. tanguticum* Maxim. ex Balf., and *R. officinale* Baill., can be used as Rhei Radix et Rhizoma. In Uyghur medicine, Tibetan medicine, and other ethnomedicine, there is a commonly-used herb named Tudahuang, which can be the root and rhizome of any of several species of the genus *Rumex* (family Polygonaceae), such as *R. patientia*, *R. japonica*, and *R. gmelinii* [25,26]. People have often confounded these other *Rumex* species with Rhei Radix et Rhizoma as they look similar. However, there is no sennoside in Tudahuang, and its purgative activity is much weaker than that of Rhei Radix et Rhizoma [27]. These substitutes and confusable varieties contain rhaponticin, a type of stilbene which is absent in Rhei Radix et Rhizoma. In this study, rhaponticin, which SHT should not contain, was found in 15 SHT samples by UHPLC-Q-TOF-MS, while it was not detected in any sample by TLC, which is the method used in *Ch.P.* for SHT identification. Due to its low sensitivity, TLC cannot detect small amounts of rhaponticin. These results clearly demonstrate the need for updating QC methods with more sensitive techniques, like HPLC, for the quality control of herbal products.

Our comprehensive quality analysis method monitored more chemical markers and could give a reasonable explanation for the failure of 11 samples to meet *Ch.P.* assay requirement regarding the total amount of emodin and chrysophanol (1.10 mg/tablet in average). Besides anthraquinone derivatives, anthrones like sennosides A and B are also accepted as important active components in Rhei Radix et Rhizoma [28,29]. The total quinone content in these 11 failed SHT samples was at a much higher average level, namely 2.57 mg/tablet. Especially for samples A1 and A2, their total amounts of emodin and chrysophanol were the lowest among 27 samples, but they contained the highest total amount of sennoside A and sennoside B. As they are also responsible for the purgative effect of Rhei Radix et Rhizoma, these two samples still can induce purging despite the low amount of emodin and chrysophanol. As the bioactivity of a Chinese medicine formula depends not only on several chemicals but groups of chemicals, it is important to monitor larger numbers of chemicals in a medicine for fair and comprehensive evaluation of an herbal medicine’s quality. The results of quality analysis of these commercial SHT samples strongly suggested this importance.

### 2.4. Effects of Coatings on SHT Evaluation

In this project, there were two types of coated tablet: sugar-coated tablet and film-coated tablet. Sugar coating increases the size and weight of the tablet by 50%–100% [30,31]. A film-coated tablet is coated with a thin layer of polymer. Compared with the sugar coating, the weight increase brought by film-coating is only 2%–3% of the tablet weight [32]. In this experiment, sugar-coated samples weighed between 329–511 mg per tablet while the weight of film-coated samples varied in a narrow range of 254 to 263 mg. All samples were extracted with their coating in order to quantify the saccharide. The different coatings caused great differences in saccharide content, especially sucrose content, which was assessed not more than 10.96 mg/tablet in film-coated SHT samples but 36.7–166.66 mg/tablet in sugar-coated SHT samples. Our control SHT sample contained little sucrose, suggesting that this component in SHT samples mainly came from coating materials. The content of sucrose varied greatly in sugar-coated tablets as it depended on the coating process.

## 3. Experimental Section

### 3.1. Chemicals and Materials

Commercial SHT products were purchased from various suppliers in mainland China. Details of the 27 samples, including 3 film-coated and 24 sugar-coated tablets, produced by 12 manufacturers are listed in Table 3. In addition, we prepared our own sample as a control using Rhei Radix et Rhizoma from Gansu, China and Scutellariae Radix from Shandong, China. These herb materials were authenticated by Professor Hu-Biao Chen from the School of Chinese Medicine, Hong Kong Baptist University, China. Voucher specimens of the herbs were deposited at the School of Chinese Medicine, Hong Kong Baptist University, China.

Reference standards of (**1**) loganic acid (Lot no. 131220); (**5**) scutellarin (130306); (**8**) apigetrin (130315); (**11**) baicalin (121128); (**12**) eriodictyol (130606); (**13**) quercetin (131120); (**14**) scutellarein (131219); (**15**) oroxyloside (121122); (**17**) wogonoside (131214); (**18**) baicalein (130119); (**19**) aloe-emodin (130425); (**21**) wogonin (140521) and (**23**) oroxylin A (130323) were provided by Chengdu Preferred Biotechnology Co. Ltd. (Chengdu, China). Reference compounds of (**3**) daidzin (131214); (**4**) sennoside B (130409); (**6**) luteoloside (130418); (**7**) sennoside A (130306); (**9**) rhaponticin (140604); (**10**) daidzein (130715); (**16**) physcion (140211); (**20**) rhein (131024); (**22**) chrysophanol (140219); (**24**) emodin (140422); (**25**) coptisine (130628); (**26**) epiberberine (130621); (**27**) jatrorrhizine hydrochloride (130318); (**28**) palmatine hydrochloride (130702) and (**29**) berberine hydrochloride (140314) were provided by Sichuan Weikeqi Biological Technology Co., Ltd. (Chengdu, China). Reference marker of (**2**) caffeic acid (130428) was provided by Chengdu Herbpurify Co., Ltd. (Chengdu, China).

The identities of the reference standards were confirmed by mass spectrometry prior to use. The purities of the reference standards were determined to be greater than 98% by UPLC-DAD analysis based on peak area normalization. Reference substances of (**30**) d-(−)-fructose (SLBB6798V), (**31**) d-(+)-glucose (070M03801V) and (**32**) sucrose (SLBF27618) were supplied by Sigma (S. Louis, MO, USA). Complanatoside A (MUST-13011217, **IS1**) from Chengdu Must Bio-Technology Co., Ltd. (Chengdu, China) and evodiamine (C-0337, **IS2**) from Hong Kong Jockey Club Institute of Chinese Medicine (Hong Kong, China) were used as internal standards.

HPLC grade acetonitrile, methanol, and formic acid were provided by RCI Labscan Limited (Bangkok, Thailand). HPLC grade ethanol was provided by Merck (Darmstadt, Germany). Hydrochloric acid (37%) was provided by VWR Chemicals (Radnor, PA, USA). Water used was purified with Millipore Milli-Q water purification system (Millipore, Bedford, MA, USA).

### 3.2. Sample Preparation

For LCMS analysis, 10 tablets from each SHT sample were ground into fine powder and passed through a 60–80 mesh filter. An accurately weighed sample (500 mg) of each powder was then extracted three times under ultrasonication with 10 mL of ethanol-water (70:30, *v*/*v*) for 30 min in a sealed 20 mL glass bottle. Due to the varied contents of different analytes in samples, some may be beyond the linear ranges, so the extracts were diluted 10×, 250× and 400× before analysis. For determination of most analytes, the extracts were diluted 10 times; for analytes No. 11, 15 and 24 in some samples, due to their higher contents in SHT, extracts were diluted 250 times; for alkaloid analytes No. 25–29, extracts were diluted 400 times. For HPLC-CAD analysis, 100 mg of the above SHT powder was extracted with 5 mL water in a sealed 20 mL glass bottle in a dry bath at 120 °C for 1.5 h. The solution was filtered, and the residue was extracted again by the same method for another one hour. The solution was filtered and combined with the first. Out of the final solution, 0.5 mL of solution from each sample was then freeze-dried before re-dissolving in 1 mL of acetonitrile–water (80:20, *v*/*v*) to prepare the sample solution.

In addition, we prepared the control sample by following the procedures in the *Chinese Pharmacopoeia 2015*. Briefly, 10 g of Scutellariae Radix was decocted with 100 mL water three times, (1.5 h, 1 h and 40 min, respectively). The decoctions were then combined and filtered. The pH value of the filtrate was adjusted to pH 1–2 by adding hydrochloric acid, and one hour later the solution was filtered again. The obtained precipitate was then washed with water to pH 5–7, heated to dryness, and ground into fine powder to get the extract of Scutellariae Radix. Rhei Radix et Rhizoma coarse powder (10 g) was refluxed with 100 mL of 30% ethanol for three times (1.5 h, 1 h and 40 min, respectively). The extracts were combined and filtered. The filtrate was then concentrated in vacuum to get a thick extract, to which 1.4 g of the dried Scutellariae Radix extract and 10 g of Rhei Radix et Rhizoma fine powder were added to make the control sample. Berberine hydrochloride and excipients were not added because berberine hydrochloride has been an identified chemical and the excipients remain unknown to us.

### 3.3. Standard Solution Preparation

The non-saccharide small molecule reference markers, as well as two internal standard compounds, were accurately weighted and dissolved in methanol to prepare a stock solution. Reference substances of three saccharides were accurately weighted and dissolved in water to prepare a stock solution. Calibration curves were obtained from standard solutions, which were prepared by appropriate dilution of the mixed standard solutions.

### 3.4. UHPLC-Q-TOF-MS Conditions

UHPLC data was collected using an Agilent 1290 Infinity UPLC system (Agilent Technologies, Santa Clara, CA, USA) equipped with a G4220A binary pump, a column compartment with a thermostat, a G4226A HiP sampler, and a degasser. Separations were conducted over an Acquity UPLC BEH C_18_ (1.7 µm, 2.1 × 100 mm, Waters, Milford, CT, USA) column at 40 °C with a gradient elution consisting of solvent A (0.1% formic acid in water) and solvent B (0.1% formic acid in acetonitrile) at a flow rate of 0.35 mL/min. The column was eluted with the following gradient program: 0–14 min, 10%–30% B; 14–22 min, 30%–37% B; 22–28 min, 37%–75% B; 28–31 min, 75%–100% B; 31–34 min, 100% B; 34–34.1 min, 100%–10% B; 34.1–36 min, 10% B. The injection volume was 2 µL.

An Agilent 6540 Q-TOF mass spectrometer (Agilent Technologies) equipped with a jet stream electrospray ionization (ESI) source was used to acquire the MS and MS/MS data in the positive and negative ionization modes. Data acquisition was controlled using MassHunterB.03 software (Agilent Technologies). The operating parameters were set as follows: nebulizing gas (N_2_) flow rate, 8 L/min; nebulizing gas temperature, 300 °C; jet stream gas flow, 8 L/min; sheath gas temperature, 350 °C; nebulizer, 45 psi; capillary, 3000 V; skimmer, 65 V; Oct RFV, 600 V; fragmentor voltage, 150 V. The peaks with the range of 100–1700 *m*/*z* were recorded.

### 3.5. HPLC-CAD Conditions

An HPLC-CAD method was used to determinate fructose, glucose and sucrose contents. A UltiMate 3000 liquid chromatography system (Dionex, Sunnyvale, CA, USA) equipped with a Dionex Corona Veo RS Charged Aerosol Detector through an Alltech Interface 35900E multichannel interface was used. The chromatographic separations were performed on an Asahipak NH_2_P-50 4E column (4.6 mm × 250 mm, Shodex, Tokyo, Japan) at a column temperature of 30 °C. The column was eluted with a mixture of water (mobile phase A) and ACN (mobile phase B) at a flow rate of 0.6 mL/min. The elution conditions were as follows: 0–17 min, 78% B; 17–21 min, 78%–62% B; 21–27 min, 62%–60% B, 27–27.1 min, 60%–78% B, followed by ten-minute balance at 78% B. The power function of CAD was 1. The injection volume was 10 µL.

### 3.6. Method Validation

The two developed UHPLC-Q-TOF-MS and HPLC-CAD methods were evaluated for linearity, sensitivity, precision, stability, and spike recovery. MS data was analyzed with MassHunter Workstation Software Quantitative, version B.06 (Agilent Technologies). HPLC-CAD data was analyzed with Chromeleon^®^ 7 Chromatography Data System, version 7.2.2.6394 (Dionex).

Stock solutions of the mixed standards were diluted to a variety of different concentrations to allow for the construction of calibration curves. At least six concentrations of each reference standard were analyzed in triplicate. The calibration curves were constructed by plotting the peak areas versus the concentrations of the corresponding constituents. The limit of detection (LOD) and limit of quantification (LOQ) values for the optimum conditions were determined at signal-to-noise ratios (S/N) of 3 and 10, respectively. The intra- and inter-day variations were used to evaluate the precision of our newly developed methods. Six independently prepared solutions of SHT were analyzed within 1 day to evaluate the intra-day variability of the optimum method. To evaluate the inter-day variability of this method, we examined the same sample twice a day over 3 consecutive days. For stability, the SHT samples were stored at room temperature and analyzed at 8, 12, 16, 24, 36, 48 h after extraction. Variations were expressed as relative standard deviations (RSDs) of the data, which were calculated using the following formula: *RSD* (%) *=* (*standard deviation*/*mean*) × 100%. A recovery test was performed to evaluate the accuracy of the optimum method by adding three different concentrations of a standard solution (i.e., low, medium and high) to SHT, which contained known quantities of the target compounds. These samples were then analyzed in parallel using our newly established method. Each experiment was conducted in triplicate at each level. The spike recoveries were calculated using the following equation: *Spike recovery* (%) *= (total amount detected − amount original)*/*amount spiked* × 100%.

## 4. Conclusions

A comprehensive and sensitive quality analysis method using UHPLC-Q-TOF-MS and HPLC-CAD was successfully established and validated for quantification of 29 non-saccharide small molecules and three saccharides in commercial SHT products. Up to 57.61% (*w*/*w*) of SHT was quantified. For the quantified components, there were 18% flavonoids, 3% anthraquinones, 3% alkaloids, and 76% saccharides which were not well-quantified in other studies. The contents of 32 analytes varied in different samples but were relatively stable between batches of the same manufacturer. There were 22 out of 27 commercial SHT samples failed the *Chinese Pharmacopoeia 2015* assays. This dissatisfactory result implicated the uneven qualities of Sanhuang tablets in the market.

However, the current *Chinese Pharmacopoeia* assays focus on only baicalin, emodin, chrysophanol and berberine hydrochloride. They are not capable of reflecting the quality of SHT as there are many other analogue compounds in the SHT. Moreover, the TLC detection of rhaponticin, the indicator of unauthorized Rhei Radix et Rhizoma, is not sensitive enough. There were 15 samples detected with the presence of rhaponticin using UPLC-Q-TOF-MS, while none was detected with rhaponticin by TLC. These results demonstrate the importance to update the QC methods. This new method provides a more fair and comprehensive quality evaluation of commercial SHT products.

## Figures and Tables

**Table 1:** The method validation in terms of linearity, sensitivity, precision, stability and accuracy for 29 analytes on UPLC-Q-TOF-MS and 3 analytes on HPLC-CAD (“N/A” = not available; for analytes 30–32, linearity ranges were calculated in mg/mL while LODs and LOQs were calculated in µg/mL).

Analyte No.	Produced Ion (*m*/*z*)	Linearity			Sensitivity		Precision (RSD, %, *n* = 6)		Stability (RSD, %, *n* = 6)	Spike Recovery (%)		
		Range (ng/mL)	Equation	R^2^	LOD (ng/mL)	LOQ (ng/mL)	Intra-Day	Inter-Day		Low	Middle	High
**1**	375.1297 [M − H]^−^	5.06–324	*y* = 401.55*x* − 990.71	0.9999	1.34	4.45	3.39	4.23	4.30	96.44	96.52	97.75
**2**	179.0350 [M − H]^−^	4.80–307	*y* = 470.18*x* − 2235.9	0.9999	1.18	3.95	3.03	3.28	2.55	95.46	99.40	102.79
**3**	415.1035 [M − H]^−^	136.56–2185	*y* = 15.357*x* − 1830.2	0.9942	1.51	5.04	2.63	4.37	4.33	100.05	98.07	102.84
**4**	861.1884 [M − H]^−^	30.47–3900	*y* = 125.48*x* − 2618.7	0.9997	1.30	4.32	3.56	2.63	2.40	100.21	104.96	96.23
**5**	461.0725 [M − H]^−^	23.28–2980	*y* = 272.92*x* − 3137.9	0.9996	1.68	5.61	2.52	3.58	3.48	98.99	95.45	104.77
**6**	447.0933 [M − H]^−^	1.41–90.3	*y* = 606.37*x* − 640.17	0.9992	0.36	1.18	1.51	2.87	2.36	103.13	103.77	96.14
**7**	861.1884 [M − H]^−^	40.00–2560	*y* = 114.98*x* − 1292.7	0.9988	1.42	4.73	1.54	3.76	2.18	97.28	103.03	99.88
**8**	431.0984 [M − H]^−^	4.68–149.8	*y* = 858.52*x* + 1059.2	0.9991	0.40	1.32	4.47	3.36	3.74	102.57	103.94	103.15
**9**	419.1348 [M − H]^−^	5.83–93.2	*y* = 365.15*x* − 529.54	0.9953	1.03	3.44	4.27	4.39	4.35	103.90	97.33	100.74
**10**	253.0506 [M − H]^−^	5.98–383	*y* = 2478.9*x* − 17151	0.9960	0.38	1.27	2.07	2.69	2.54	104.94	104.17	98.81
**11**	445.0776 [M − H]^−^	135.00–8640	*y* = 430.59*x* + 14794	0.9994	1.38	4.61	3.17	2.53	2.97	102.67	101.52	97.84
**12**	287.0561 [M − H]^−^	3.49–223.5	*y* = 1247.9*x* − 822.3	0.9997	1.10	3.38	4.44	3.69	4.70	95.92	97.71	99.63
**13**	301.0354 [M − H]^−^	18.28–585	*y* = 540.03*x* − 9261.2	0.9975	2.22	7.40	3.15	3.73	3.18	104.89	104.79	96.59
**14**	285.0405 [M − H]^−^	21.72–2780	*y* = 268.75*x* + 6706	0.9998	2.01	6.71	2.05	4.00	4.13	94.89	99.00	102.75
**15**	459.0933 [M − H]^−^	29.84–3820	*y* = 307.34*x* + 681.54	0.9998	1.88	6.26	3.94	3.73	3.84	101.31	105.11	100.15
**16**	283.0612 [M − H]^−^	36.09–4620	*y* = 32.549*x* − 164.18	0.9988	3.09	10.29	3.16	3.75	3.81	99.17	99.95	103.28
**17**	459.0933 [M − H]^−^	30.47–3900	*y* = 650.05*x* + 11427	0.9998	0.94	3.14	3.27	2.48	3.72	96.81	101.72	103.69
**18**	269.0455 [M − H]^−^	33.2–2125	*y* = 293.64*x* − 1480.4	0.9951	2.13	7.09	4.61	4.09	4.35	96.10	96.77	94.89
**19**	269.0455 [M − H]^−^	43.01–5505	*y* = 289.26*x* + 4789.9	0.9999	0.45	1.51	3.58	4.60	4.75	101.45	105.02	102.38
**20**	283.0248 [M − H]^−^	19.41–2485	*y* = 819.28*x* − 17041	0.9963	1.24	4.12	3.29	2.42	4.42	103.83	97.74	99.60
**21**	283.0612 [M − H]^−^	16.80–2150	*y* = 1295.6*x* + 16017	0.9994	0.76	2.54	2.54	4.56	4.81	95.46	94.62	97.75
**22**	253.0506 [M − H]^−^	42.66–5460	*y* = 449.73*x* + 2250.9	0.9994	2.53	8.44	3.89	3.77	4.18	100.14	95.07	96.08
**23**	283.0612 [M − H]^−^	5.31–680	*y* = 495.34*x* + 2329	0.9998	1.48	4.93	2.63	4.16	3.31	105.21	94.93	98.93
**24**	269.0455 [M − H]^−^	93.91–3005	*y* = 1565.2*x* + 261164	0.9977	0.62	2.08	4.09	3.16	4.65	104.15	101.50	102.61
**25**	320.0917 [M + H]^+^	10.50–672	*y* = 1781.3*x* − 4014.8	0.9996	0.85	2.83	3.86	2.48	4.68	94.88	102.17	103.99
**26**	336.1230 [M + H]^+^	4.95–79.2	*y* = 1757*x* − 9296.5	0.9992	0.47	1.55	4.36	4.62	4.44	102.14	101.39	99.42
**27**	338.1387 [M + H]^+^	3.67–470	*y* = 11036*x* − 13846	0.9991	0.67	2.23	4.80	2.58	3.04	102.22	99.14	100.31
**28**	352.1543 [M + H]^+^	0.93–119.4	*y* = 62748*x* + 1138.7	0.9995	0.13	0.45	2.48	4.46	3.30	104.86	96.79	100.75
**29**	336.1230 [M + H]^+^	42.19–5400	*y* = 841.23*x* − 17813	0.9992	1.91	6.36	4.61	4.26	4.01	99.52	101.44	96.01
**30**	N/A	0.23–8.27	*y* = 18.08*x* + 4.31	1.00	1.24	4.13	4.09	4.75	2.95	94.35	104.94	103.92
**31**	N/A	0.25–3.94	*y* = 15.05*x* + 5.80	0.99	1.84	6.15	4.75	4.96	4.43	94.17	98.78	99.02
**32**	N/A	0.47–7.53	*y* = 12.90*x* + 12.38	0.99	2.27	7.56	2.38	2.58	3.12	104.28	101.11	95.01

**Table 2:** Contents (µg/tablet) of 32 analytes in 27 SHT commercial samples and self-made sample (“ND” = not detected; “N/A” = not available).

**Analyte No.**	**A1**	**A2**	**B1**	**C1**	**C2**	**D1**	**D2**	**E1**	**E2**	**E3**	**E4**	**E5**	**E6**	**E7**
**1**	1.79	1.00	ND	ND	ND	ND	ND	ND	ND	ND	ND	ND	ND	ND
**2**	4.26	5.15	4.11	1.71	2.31	1.91	1.98	6.52	3.10	3.68	1.62	1.73	1.72	1.20
**3**	181.74	192.85	100.41	ND	ND	49.58	353.93	536.31	104.52	91.81	78.77	88.80	81.96	47.45
**4**	1279.11	860.85	151.48	ND	12.31	13.44	19.55	6.88	ND	5.45	8.58	ND	ND	ND
**5**	79.74	121.41	78.61	107.21	111.92	103.71	112.29	146.44	140.75	147.09	141.05	108.64	129.59	97.01
**6**	ND	ND	1.19	0.56	ND	0.54	1.33	1.46	0.76	1.55	1.21	0.71	1.66	0.56
**7**	3367.27	2197.00	362.33	ND	47.07	61.20	76.91	11.48	13.79	7.29	ND	ND	5.31	ND
**8**	18.99	19.25	8.34	ND	ND	5.28	6.43	7.84	6.02	5.27	6.56	4.72	6.01	3.08
**9**	ND	1.41	ND	ND	ND	ND	ND	8.90	6.57	2.57	2.16	2.72	2.32	3.50
**10**	ND	1.12	1.22	ND	ND	0.28	ND	ND	ND	ND	ND	ND	0.35	ND
**11** ^a^	17.97	17.25	24.71	12.61	14.53	18.94	61.75	21.50	25.39	22.54	15.48	21.71	22.74	20.24
**12**	3.78	5.77	8.59	ND	ND	2.11	6.90	2.40	2.70	1.79	3.14	1.59	2.77	1.05
**13**	4.69	3.22	3.15	6.17	5.19	6.00	8.03	6.62	5.52	4.95	8.66	6.54	5.26	5.34
**14**	4.59	ND	13.19	17.13	11.53	14.16	14.25	17.65	24.68	15.29	21.15	17.15	18.44	12.44
**15** ^a^	2.60	2.42	4.32	2.32	4.36	2.62	9.25	3.63	4.05	2.86	1.80	4.00	3.89	2.31
**16**	202.70	184.88	224.31	918.55	2991.21	348.89	384.46	683.47	458.18	761.59	712.93	477.11	573.91	519.79
**17** ^a^	0.17	0.17	0.17	0.86	7.72	0.30	0.31	0.58	0.38	0.64	0.60	0.38	0.46	0.42
**18**	362.08	389.73	336.03	372.06	972.33	771.57	1966.53	661.36	727.25	684.52	751.46	424.68	520.21	433.45
**19**	43.52	96.41	60.29	12.74	28.69	61.01	47.21	39.63	49.48	54.10	52.88	44.15	70.25	21.78
**20**	88.74	89.67	227.31	42.36	43.51	218.35	196.33	107.70	114.96	115.98	117.79	108.63	167.23	57.76
**21**	35.47	52.08	72.74	116.12	492.97	98.24	110.89	48.18	50.30	73.31	70.15	30.46	39.99	37.33
**22**	33.49	63.30	54.25	34.27	142.81	47.74	82.13	40.66	81.04	45.46	47.72	24.85	35.61	28.90
**23**	19.11	34.80	29.34	45.86	154.57	76.07	97.86	56.20	53.30	69.62	68.55	45.23	45.50	39.66
**24** ^a^	0.75	0.40	3.55	3.10	6.53	1.12	4.46	0.30	1.49	1.05	1.04	0.74	1.19	1.19
**25**	ND	ND	55.55	ND	ND	49.67	ND	ND	ND	ND	ND	ND	ND	ND
**26**	ND	3.01	ND	ND	ND	6.10	ND	ND	ND	5.23	ND	ND	ND	ND
**27**	9.70	14.92	13.49	10.38	18.91	9.67	ND	35.16	21.89	13.22	19.26	16.94	19.55	18.85
**28**	ND	ND	ND	ND	ND	ND	ND	ND	ND	ND	ND	ND	ND	ND
**29** ^a^	3.57	4.20	3.55	5.46	5.03	5.56	5.88	3.02	5.51	2.08	3.13	4.17	4.80	5.11
**30** ^a^	8.65	2.70	6.96	3.26	13.76	44.55	34.74	23.15	44.24	29.26	12.14	18.76	18.71	15.45
**31** ^a^	12.39	6.29	6.08	25.88	15.16	39.88	27.81	19.87	42.34	27.93	11.12	16.40	15.95	12.96
**32** ^a^	ND	2.18	10.96	73.01	90.45	146.16	141.47	74.61	93.56	86.55	77.97	87.18	84.66	75.54
Flavonoids (%)	8.37	8.14	11.36	3.91	6.54	4.55	14.63	5.92	7.21	6.74	4.65	5.68	6.48	5.07
Anthraquinones (%)	2.25	1.53	1.76	0.98	2.26	0.37	1.04	0.26	0.52	0.51	0.48	0.30	0.47	0.39
Alkaloids (%)	1.40	1.66	1.38	1.30	1.17	1.11	1.16	0.66	1.29	0.52	0.77	0.89	1.12	1.10
Saccharides (%)	8.20	4.41	9.13	24.26	27.54	45.63	40.34	25.59	42.01	35.67	24.76	25.90	27.67	22.30
Total quantified content (%)	20.22	15.75	23.63	30.44	37.51	51.66	57.17	32.44	51.03	43.44	30.67	32.76	35.74	28.86
**No.**	**F1**	**F2**	**F3**	**F4**	**F5**	**F6**	**F7**	**G1**	**H1**	**I1**	**J1**	**K1**	**L1**	**Control**
**1**	ND	ND	ND	ND	ND	ND	ND	ND	ND	ND	ND	ND	ND	ND
**2**	1.78	1.86	1.31	2.46	ND	1.96	1.16	3.37	1.18	1.10	1.94	1.57	1.12	5.61
**3**	28.94	36.83	39.09	ND	ND	ND	ND	34.75	ND	266.14	90.75	249.30	65.36	149.00
**4**	39.62	29.82	27.04	39.41	26.99	24.81	21.02	ND	ND	ND	5.54	50.08	ND	18.09
**5**	113.38	171.73	147.27	131.05	139.25	130.27	134.66	118.10	166.47	116.07	90.17	131.60	178.82	92.82
**6**	ND	ND	0.46	0.38	ND	ND	ND	4.12	0.34	0.72	0.35	1.35	1.58	3.32
**7**	127.49	116.73	122.54	183.99	121.73	104.00	116.15	16.32	ND	ND	16.20	407.50	8.99	88.81
**8**	ND	ND	ND	ND	ND	ND	ND	3.87	1.80	1.77	3.09	26.24	5.06	15.94
**9**	ND	1.56	ND	3.66	2.16	1.44	4.14	ND	ND	ND	2.08	ND	9.15	ND
**10**	ND	ND	ND	ND	ND	ND	ND	ND	ND	ND	ND	2.67	0.37	ND
**11** ^a^	22.07	13.77	19.46	22.41	18.69	19.05	20.99	13.01	19.85	13.72	22.07	16.20	27.32	18.06
**12**	ND	ND	ND	1.79	1.07	ND	1.06	ND	ND	0.80	1.16	1.02	5.16	6.94
**13**	ND	ND	5.85	ND	5.54	5.45	ND	9.28	4.00	4.83	5.30	5.18	5.32	17.08
**14**	16.91	18.63	16.66	24.70	21.94	23.52	22.79	13.12	14.60	ND	17.35	17.21	23.41	40.60
**15** ^a^	3.19	1.62	2.73	3.77	3.08	2.93	3.25	6.72	3.17	3.56	2.76	2.88	4.78	1.01
**16**	667.87	654.84	1195.25	743.87	434.08	1115.16	777.99	512.77	437.07	2772.60	754.14	664.23	1071.51	379.24
**17** ^a^	0.61	1.46	1.04	0.60	0.35	0.87	0.62	31.48	0.93	6.66	0.67	0.50	2.32	2.56
**18**	553.28	374.81	536.77	426.99	565.08	550.36	505.21	1652.22	710.34	835.91	321.44	484.94	819.93	471.95
**19**	93.40	95.98	107.27	101.19	94.41	111.82	122.06	50.44	36.85	36.35	25.84	56.72	90.46	94.69
**20**	238.50	243.70	250.81	259.95	230.23	252.24	271.63	15.33	41.20	83.25	59.61	135.24	267.85	489.38
**21**	55.74	65.70	61.12	51.30	47.99	64.38	62.29	2444.05	16.11	408.15	38.95	62.02	86.38	268.72
**22**	23.60	21.64	25.42	58.58	38.51	26.50	45.69	235.95	90.88	113.55	13.55	50.24	178.54	130.01
**23**	26.93	17.36	24.88	31.74	23.43	23.92	29.85	585.63	39.03	171.83	24.18	39.45	335.56	45.15
**24** ^a^	4.57	2.68	3.30	3.89	6.95	3.32	3.61	3.72	3.75	0.64	5.06	1.26	1.73	1.50
**25**	ND	ND	ND	ND	ND	ND	ND	ND	ND	ND	ND	ND	ND	N/A
**26**	ND	ND	ND	5.53	ND	5.63	ND	ND	ND	ND	ND	ND	ND	N/A
**27**	60.56	91.12	60.72	68.16	72.59	247.30	57.05	20.10	137.83	17.15	81.82	18.62	9.17	N/A
**28**	ND	ND	ND	ND	174.52	17.41	ND	13.12	1.49	ND	ND	ND	ND	N/A
**29** ^a^	1.51	6.82	3.91	5.25	4.94	19.67	3.56	5.16	6.62	3.43	10.72	4.42	4.85	N/A
**30** ^a^	16.49	26.02	24.07	15.06	15.21	15.78	14.99	40.17	40.92	24.43	24.97	37.63	29.73	21.93
**31** ^a^	16.37	24.58	22.54	11.32	14.85	14.83	14.55	38.67	40.98	23.21	23.59	34.49	29.19	16.36
**32** ^a^	89.10	67.37	100.01	108.03	106.43	85.94	86.97	40.46	36.70	92.68	166.66	93.27	54.53	ND
Flavonoids (%)	6.09	3.84	5.54	5.60	5.07	5.19	5.76	10.98	7.57	6.38	5.83	4.66	8.12	12.04
Anthraquinones (%)	1.32	0.84	1.16	1.08	1.75	1.09	1.12	0.89	1.32	0.90	1.32	0.59	0.76	1.43
Alkaloids (%)	0.36	1.51	0.91	1.09	1.15	4.38	0.81	1.02	2.06	0.85	2.41	1.00	1.10	N/A
Saccharides (%)	27.87	25.84	33.78	27.43	30.21	25.59	26.23	23.36	36.07	34.74	48.05	37.43	25.63	20.26
Total quantified content (%)	35.63	32.04	41.39	35.19	38.18	36.24	33.92	36.24	47.02	42.87	57.61	43.69	35.61	33.73

^a^ calculated in mg/tablet.

**Table 3:** Basic information of the 27 commercial SHT samples.

No.	Manufacturer	Batch No.	Coating Material	Average Weight (mg)
**A1**	Manufacturer A	20130601	Film	256.40
**A2**		33140101	Film	253.50
**B1**	Manufacturer B	131201	Film	262.80
**C1**	Manufacturer C	130402	Sugar	433.475
**C2**		140404	Sugar	477.95
**D1**	Manufacturer D	130101	Sugar	505.40
**D2**		130501	Sugar	505.78
**E1**	Manufacturer E	130102	Sugar	459.60
**E2**		130502	Sugar	428.83
**E3**		131001	Sugar	402.93
**E4**		131002	Sugar	408.83
**E5**		131004	Sugar	472.4
**E6**		131102	Sugar	431.23
**E7**		130901	Sugar	466.15
**F1**	Manufacturer F	130402	Sugar	437.70
**F2**		130604	Sugar	456.58
**F3**		130701	Sugar	434.10
**F4**		130906	Sugar	490.10
**F5**		131001	Sugar	451.80
**F6**		130705	Sugar	455.50
**F7**		130903	Sugar	444.25
**G1**	Manufacturer G	131003	Sugar	510.75
**H1**	Manufacturer H	131001	Sugar	328.80
**I1**	Manufacturer I	140201	Sugar	403.98
**J1**	Manufacturer J	130604	Sugar	447.95
**K1**	Manufacturer K	20130903	Sugar	441.90
**L1**	Manufacturer L	131002	Sugar	442.65
